# On the taxonomic affinity of *Albiziacarbonaria* Britton (Leguminosae, Caesalpinioideae-mimosoid clade)

**DOI:** 10.3897/phytokeys.205.82288

**Published:** 2022-08-22

**Authors:** Erik J. M. Koenen

**Affiliations:** 1 Evolutionary Biology & Ecology, Université Libre de Bruxelles, Faculté des Sciences, Campus du Solbosch - CP 160/12, Avenue F.D. Roosevelt, 50, 1050 Bruxelles, Belgium Université Libre de Bruxelles Bruxelles Belgium

**Keywords:** *Albizia* sect. *Arthrosamanea*, polyads, *Pseudosamanea*, taxonomy

## Abstract

Recent phylogenomic analyses placed *Albiziacarbonaria* Britton as the sister-group of the two currently known species of *Pseudosamanea* Harms, clearly outside AlbiziasectionArthrosamanea (Britton & Rose) Barneby & J.W. Grimes where it has until now been included. Its morphological similarities to *Pseudosamanea* are discussed, including characteristics of the polyad, and it is concluded that the species is compatible with the generic description of that genus except for its much more finely divided leaves with smaller leaflets, and its smaller flowers and fruits. Since these are merely quantitative differences, the species can readily be accommodated in *Pseudosamanea*. The new combination *Pseudosamaneacarbonaria* (Britton) E.J.M. Koenen is made, and a diagnosis distinguishing it from the other two species of *Pseudosamanea* is presented.

## Introduction

**Figure 1.**
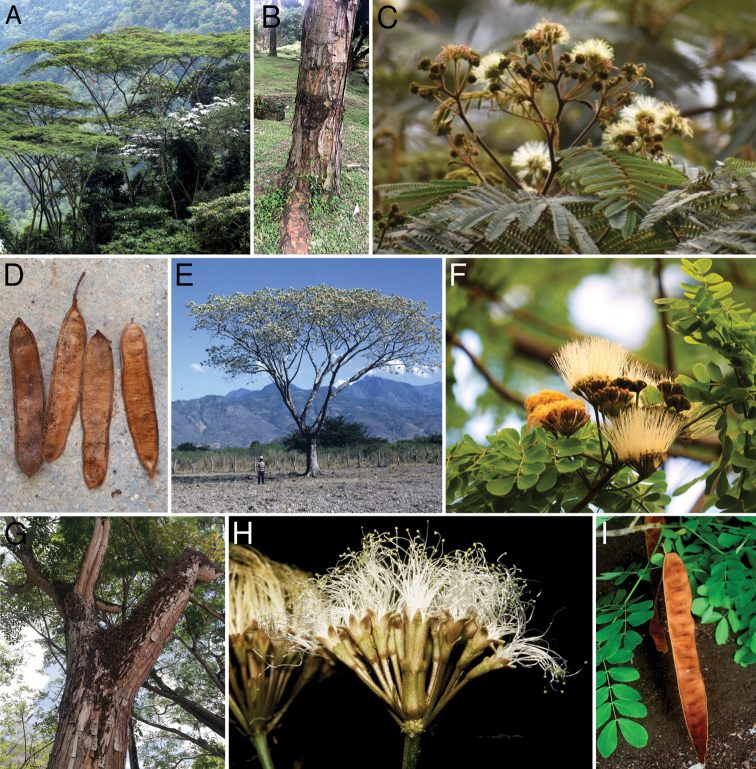
**A–D***Pseudosamaneacarbonaria* (Britton) E.J.M. Koenen **A** habit **B** trunk with exfoliating bark **C** inflorescences **D** pods **E–I***Pseudosamaneaguachapele***E** habit **F** inflorescences **G** trunk with exfoliating bark **H** close-up of strongly heteromorphic inflorescence typical of the genus **I** pod **A** Bioexploradores Farallones **B** Karen Osorio **C** Juan Manuel de Roux **D** Juan Carlos Delgado Madrid **E, H** Colin Hughes **F** Cynthia Tercero **G** Bribrábrico **I** Daniel H Janzen **A, B, C, D, F, G, I** from https://www.gbif.org, distributed under a Creative Commons BY-NC-SA 3.0 License.

Recent phylogenomic analysis (Ringelberg et al. 2022) placed *Albiziacarbonaria* Britton as the sister-group to *Pseudosamenea* Harms, separate from other Neotropical species of *Albizia* Durazz. that Barneby and Grimes (1996) placed in their section Arthrosamanea (Britton & Rose) Barneby & J.W. Grimes. Given the finely divided microphyllidious foliage and slender inflorescences of *A.carbonaria*, with much smaller flowers than those of the macrophyllidious species of *Pseudosamanea*, this may at first sight seem an unexpected phylogenetic relationship. However, on closer inspection, the similarities between *A.carbonaria* and *Pseudosamanea* are immediately apparent (Fig. 1) from the umbellate capitula with distinctly pedicellate peripheral flowers and a single enlarged sessile central flower (Fig. 1H), the ferrugineous indumentum on the twigs, leaf axes, peduncles, flowers and pods, the distinctive exfoliating bark (Fig. 1B, G), and the plano-compressed fruits and papery texture of the pod valves (Fig. 1D, I). In particular, the strongly dimorphic nature of the capitula, with only the much larger central flower being sessile, is unlike any of the species in Albiziasect.Arthrosamanea, but is effectively a miniature version of the capitula of *Pseudosamaneaguachapele* (Kunth) Harms (Fig. 1C, F, H).

*Albiziacarbonaria* clearly differs from *Ps.guachapele* and *Pseudosamaneacubana* (Britton & Rose) Barneby & J.W. Grimes in having more numerous and smaller leaflets (i.e. microphyllidious instead of macrophyllidious, Barneby and Grimes 1996), that are otherwise similar by being discolorous and in possessing a single midrib with pinnate secondary venation which also differs from the typically palmate or palmately-pinnate venation of leaflets of Albiziasect.Arthrosamanea. The stipitate, indehiscent or tardily dehiscent fruits of *A.carbonaria* are more similar to those of *Ps.cubana*, while those of *Ps.guachapele* dehisce actively along the ventral suture while the pods are still on the tree. Pods in all three species have the same characteristic ferrugineous indumentum. Thus, the flowers, leaflets and pods of *A.carbonaria* are merely quantitatively different from those of *Pseudosamanea*, being (much) smaller relative to *Ps.guachapele* and *Ps cubana*.

## Evidence from polyad grain numbers

Based on its 32-celled polyads (Niezgoda and Nevling 1979a: figs 1 and 2; confirmed by my own observation, *G.P. Lewis 3862*), *A.carbonaria* was previously excluded from *Albizia* by Niezgoda and Nevling (1979a) who suggested it was preferable to restrict (Neotropical) *Albizia* to those species with 16-celled polyads. They placed *A.carbonaria* in *Pithecellobium* Mart., generating the only homotypic synonym of the species that I am aware of, which is somewhat remarkable for a species of the ingoid clade that was first described nearly a century ago, but all other authors (Nielsen 1985; Barneby and Grimes 1996; Rico-Arce et al. 2008) included it without question in *Albizia*. It is notable that *Pithecellobium*, under its current circumscription, is reported as having consistently 16-celled polyads, while Albiziasect.Arthrosamanea has either 16- or (28-)32-celled polyads (Guinet and Grimes 1997). Unfortunately, Guinet and Grimes (1997) described the characteristics of the pollen by genus, and failed to list which *Albizia* species have 32-celled polyads. However, Niezgoda and Nevling (1979a) compared the 32-celled polyads of *Pithecellobiumdaulense* Spruce ex Benth., which is (somewhat ironically) now a synonym of *Albiziapistaciifolia* (Willd.) Barneby & J.W. Grimes, with 16-celled polyads of *Albiziaretusa* Benth. However, no voucher is cited for the *P.daulense* polyad in that publication, but in Niezgoda et al. (1983) the same species is included, with voucher *Maguire 56150* which has since been re-identified as *Enterolobiumgummiferum* J.F. Macbr. in NY (specimen consulted online, http://sweetgum.nybg.org/science/vh/, Barcode: 00924167). Further reason to doubt that *A.pistaciifolia* has 32-celled polyads is that in mimosoid species with polyads the fruit is usually pollinated by a single polyad (Niezgoda and Nevling 1979a; see also Banks et al. 2010), suggesting that species with 16-celled polyads should have a maximum of 16 seeds per fruit, and *A.pistaciifolia* is described as having 11–15 seeded fruits (Barneby and Grimes 1996). If *A.pistaciifolia* indeed has 16-celled polyads, it is possible that when *A.carbonaria* is excluded, Albiziasect.Arthrosamanea will be fundamentally characterized by 16-celled polyads. This seems likely for two reasons: (1) all taxa whose pollen has been studied in the genera *Hydrochorea* Barneby & J.W. Grimes (sensu Soares et al. 2022), *Jupunba* Britton & Rose and *Punjuba* Britton & Rose (i.e. the “Abarema alliance” of Barneby and Grimes 1996), that together form the sister-group of Albiziasect.Arthrosamanea in the Jupunba clade (sensu Koenen et al. 2020; Ringelberg et al. 2022), consistently have 16-celled polyads (Guinet and Grimes 1997); (2) all species included by Barneby and Grimes (1996) in Albiziasect.Arthrosamanea have fruits that are usually c. 11–15 seeded (although occasionally up to 18-seeded in *Albiziainundata* (Mart.) Barneby & J.W. Grimes, which accordingly appears to have 16(-20?)-celled polyads, see Vossler 2019: fig. 2c & e), except for *A.carbonaria* that has 20–26-seeded fruits (Barneby and Grimes 1996).

*Pseudosamaneaguachapele* has 32-celled polyads (Niezgoda and Nevling 1979b; Guinet and Grimes 1997); therefore those of *A.carbonaria* are compatible with *Pseudosamanea*. The polyads of *Ps.cubana* are reported to be 24-celled (Guinet and Grimes 1997), but the fruit is described as 24–30 seeded (Barneby and Grimes 1996), casting doubt on the reported number of grains per polyad, or suggesting that the species is perhaps variable in grain number per polyad. Given the variation in the number of cells per polyad within several genera as well as within species or even individuals (e.g. *Leucochloron* Barneby & J.W. Grimes; Guinet and Grimes 1997), this character has been viewed as unimportant for generic delimitation in recent decades, despite mention by Guinet and Grimes (1997) that several groups are invariant in this regard. Phylogenetic studies are now revealing that polyad grain number may be more useful than previously thought. For example, the Calliandra clade (sensu Koenen et al. 2020) comprising *Acaciella* Britton & Rose, *Calliandra* Benth. and *Afrocalliandra* E.R. Souza & L.P. Queiroz has consistently 7 or 8-celled polyads, which is unusual in the ingoid clade where species usually have ≥ 16 cells per polyad. Similarly, I suggest that the entire Jupunba clade sensu Koenen et al. (2020) may be fundamentally 16-celled, and many of the genera that Guinet and Grimes (1997) described as having > 16 cells per polyad or of variable polyad number are placed in the Inga clade of Koenen et al. (2020; see also Ringelberg et al. 2022). All this suggests that the utility of polyad grain number should be re-evaluated in the light of recent phylogenomic evidence. In conclusion, the decision of Niezgoda and Nevling (1979a) to exclude *A.carbonaria* from *Albizia* based on the grain number per polyad was correct and in line with the other morphological differences discussed here; however, they did not place it in the correct genus. Instead, the 32-celled polyads provide further evidence in support of placing the species in *Pseudosamanea*.

## Inclusion of *A.carbonaria* in *Pseudosamanea*

An important character used by Barneby and Grimes (1996) to define *Pseudosamanea* is inflorescences axillary to coeval leaves (although in *Ps.guachapele* flowers often develop before the leaves). The inflorescences of *A.carbonaria* appear somewhat similar to those typically found in Albiziasect.Arthrosamanea and many other mimosoid genera where the capitula form a compound inflorescence resembling a pseudo-raceme or a complex panicle of pseudo-racemes with suppression or early shedding of leaves. However, it is questionable whether the description of the inflorescences of *Pseudosamanea* by Barneby and Grimes (1996) is completely accurate, as specimens at K (*S.C. Sant’Ana 1023* and *C.E. Hughes 753*) have short compound pseudo-racemose inflorescences, comprising multiple umbellate capitula arising from a leaf axil (rather than only singly as described for *Ps.guachapele* by Barneby and Grimes 1996), with the subtending leaves being caducous. Another key feature of the inflorescences of *Pseudosamanea* is that the meristem at the apex of reproductive branches continues to grow (i.e. is indeterminate), producing new leaves below which the fruits develop. Unlike in other species of Neotropical *Albizia*, this is clearly also the case in *A.carbonaria*, see e.g. *E. Suclli & J. Farfán 1258* – NY, Barcode 1300235; *J. Leon 4372* – F, https://collections-botany.fieldmuseum.org/, Catalog number 1578909), providing evidence that the inflorescences of *A.carbonaria* are not qualitatively different from *Pseudosamanea*, while being unlike the efoliate pseudoracemes typically found in Albiziasect.Arthrosamanea where the meristem is not continuous.

Barneby and Grimes (1996: 226) noted that “among related albizias with multifoliolate leaves, *A.carbonaria* is notable for the dorsally pallid leaflets, the dense golden indumentum of the inflorescence, the distinctly pediceled flowers, and the papery tomentulose pod.” All these characters distinguishing it from other Albiziasect.Arthrosamanea, are in agreement with the generic description of *Pseudosamanea* (sensu Barneby and Grimes 1996). *Albiziacarbonaria* differs from *Pseudosamanea* as delimited by Barneby and Grimes (1996) in the dimensions and number of leaflets per leaf, flower dimensions and inflorescence structure, but these are minor quantitative differences that do not constitute sufficient reason for not including the species in *Pseudosamanea*, an option which would require segregation of a new monotypic genus. I therefore propose to include the species in *Pseudosamanea* and hence the new combination is made:

### Pseudosamanea carbonaria

Taxon classificationPlantaeFabalesLeguminosae

(Britton) E.J.M. Koenen comb.nov.

E90DBB00-EE9D-5141-972F-338B7B0E23DC

urn:lsid:ipni.org:names:77303801-1

Fig. 1A–D

Albizia carbonaria Britton, Sci. Surv. Porto Rico & Virgin Islands 6: 348. 1926. Basionym
Albizia malacocarpa Standl. ex Britton & Rose, N. Amer. Fl. 23: 44. 1928. - Types: *Calderón 2024* (lectotype: NY, [NY00001767], chosen here; isolectotypes: US, [US00000471], GH [GH00069258]); *Williams 952* (paratype: NY, [NY01300065]).
Albizia sumatrana Steenis, in Encycl. Ned.-Ind. ed. 2, Suppl. Vol. vi. 864. 1931. - Type: *Keuchenius s.n.* (holotype: BO, isotypes: A, [A00058480]; A, [A00058481]; BO).
Pithecellobium carbonarium (Britton) Niezgoda & Nevling, Phytologia 44: 310. 1979.
Albizia filicina Standl. & L.O. Williams ex L. Holdridge & Poveda, Arboles de Costa Rica 1: 134. 1975. nomen nudum.

#### Type.

*C.L. Bates s.n.* (holotype: NY, [NY00001778]; isotype: K!, [K000528017]).

*Pseudosamaneacarbonaria* can easily be distinguished from the other two currently known species of *Pseudosamanea* by having 8–13 pairs of pinnae and (18–)20–30 leaflet pairs, compared to 3–6 pairs of pinnae and 5–8 leaflet pairs, as well as leaflet size (the larger ones 4.5–8 mm long vs. 23–50 mm) and flower size (corolla of peripheral flowers (4–)4.4–6.4 mm long vs 9.5–11 mm in *Ps.guachapele* and 11–13 mm in *Ps.cubana*; the stamen filaments 13–16.5 mm vs. 41–45 mm in *Ps.guachapele* and 25–27 mm in *Ps.cubana*) and fruit size 7–12 × 1.5–2.35 cm long (excluding the stipe) vs. c. 12–22 × 2–4.5 cm in the other two species. [All measurements taken from Barneby and Grimes (1996)].

#### Representative material studied.

*Ps.carbonaria*: Colombia: *G.P. Lewis 3862* (K, 2 sheets, fls & frts), R.T. Pennington *694* (K, fls), *H.P. Fuchs & L. Zanella 22388* (K, fls); Peru: *A. Daza & T.D. Pennington 16353* (K, fls & frts), *E. Suclli & J. Farfán 1258* (K, 2 sheets, fls & frts).

*Ps.guachapele*: Mexico: *C.E. Hughes* 665 (K, frts), *E.A. Pérez-García* 1035 (K, fls); Honduras: *C.E. Hughes & B.T. Styles 117* (K, frts), *C.E. Hughes 753* (K, fls). Guatemala: *D.J. Macqueen 68* (K, fls & frts), *C.E. Hughes 1103* (K, fls).

To identify the species of the genus *Pseudosamanea*, the following identification key, based on that of Barneby and Grimes (1996), but with an additional identification step to include *Ps.carbonaria*, can be used:

### Key to the species of *Pseudosamanea*

**Table:** 

1	Leaves with 8–13 pairs of pinnae and (18–)20–30 pairs of leaflets per pinna	***Ps.carbonaria***
–	Leaves with 3–4 pairs of pinnae and 5–8 pairs of leaflets per pinna	**2**
2	Pedicel of outer peripheral flowers 11–22 mm; pods sessile 10–20-seeded; SE Mexico to Venezuela and NE Peru	***Ps.guachapele***
–	Pedicel of outer peripheral flowers 4–6.5 mm; pods stipitate 24–30-seeded; Cuba	***Ps.cubana***

With the addition of *Pseudosamaneacarbonaria*, the genus now comprises three species with native distribution from S Mexico to N Peru and in Cuba (*Ps.cubana*, endemic), occurring in seasonally dry deciduous forest and gallery forest up to 1000 m (*Ps.guachapele*), moist upland forest up to 1800 m (*Ps.carbonaria*), and palm savannas and along watercourses below 50 m (*Ps.cubana*). Two species, *Ps.carbonaria* and *Ps.guachapele*, are cultivated including outside their native range on the Atlantic coast of Brazil, Cameroon (*Ps.guachapele*) and Indonesia (*Ps.carbonaria*). While *Ps.guachapele* is naturally widespread across the range of the genus (except for Cuba), the native range of *Ps.carbonaria* is not known with certainty, but is presumed to be from Colombia to Panama and Venezuela, and it is introduced as a shade tree in coffee plantations in Central America, the Caribbean, Peru and SE Brazil (Barneby and Grimes 1996).

## Supplementary Material

XML Treatment for Pseudosamanea carbonaria
